# A holistic approach is needed for women with an inflammatory arthritis in the different phases around pregnancy; the results of the CAPRI study

**DOI:** 10.3389/fgwh.2024.1504095

**Published:** 2025-01-29

**Authors:** A. J. van Steensel – Boon, H. M. Wintjes, E. Röder, H. T. W. Smeele, L. J. C. Kranenburg - van Koppen, R. J. E. M. Dolhain, L. F. Perez-Garcia

**Affiliations:** Department of Rheumatology, Erasmus University Medical Center, Rotterdam, Netherlands

**Keywords:** inflammatory arthritis, pregnancy, reproductive rheumatology, patient satisfaction, specialized nurse, integrated care pathway

## Abstract

**Introduction:**

Women with inflammatory arthritis (IA) face significant challenges throughout preconception, pregnancy, and postpartum phases, including concerns about disease management and medication safety. The Reproductive Rheumatology care pathway at Erasmus University Medical Center integrates specialized care from rheumatologists and specialized nurses to address both medical, nursing, practical and emotional needs during these phases. This study evaluates patient satisfaction, identifies unmet needs, and explores opportunities for enhancing support within this integrated care model.

**Methods:**

This was a cross-sectional study. We designed a customized questionnaire for women 18 years and older who were treated following the Reproductive Rheumatology care pathway and had given birth between 2019 and 2021. These women were invited to fill in the questionnaire. The survey assessed satisfaction with care, challenges experienced, and information needs across preconception, pregnancy, and postpartum phases. Descriptive statistics and paired *t*-tests were used for data analysis.

**Results:**

Participants reported high satisfaction with care, rating rheumatologists an average of 8.8/10 and specialized nurses 9.2/10. While 78.9% experienced no major issues, some faced problems such as managing disease flares and difficulties around conception. Information needs varied by phase: preconception needs focused on medication safety and fertility, while pregnancy and postpartum concerns included disease management and emotional support. Specialized nurses were pivotal in offering personalized care and practical advice.

**Conclusion:**

The integrated Reproductive Rheumatology care pathway effectively supports women with IA through their reproductive journey. Despite high satisfaction, improvements could be made in personalized care and addressing challenges related to confidence and help acceptance. Future research should investigate the long-term impact of such care pathways on reproductive outcomes and patient well-being.

## Introduction

Recent advancements in inflammatory arthritis (IA), such as early diagnosis, prompt initiation of disease-modifying antirheumatic drugs (DMARDs), including tumor necrosis factor (TNF) inhibitors, and a treat-to-target (T2T) approach aiming for remission, have led to improved patient outcomes and quality of life (1). Nonetheless, for women diagnosed with IA and a wish to conceive (or a pregnancy) represents a challenge because it is known that IA significantly impacts fertility and pregnancy outcomes (2, 3). Specialized care remains crucial to navigate the complexities around conception, pregnancy, and the postpartum phase (4).

At the Department of Rheumatology at Erasmus University Medical Center, women diagnosed with IA who wish to conceive (or are already pregnant) have access to an established comprehensive “Reproductive Rheumatology” care pathway. A care pathway is a multidisciplinary plan that outlines the essential steps in the care of patients with a specific clinical problem, aimed at improving the quality and coordination of care (5). The Reproductive Rheumatology care pathway at Erasmus University Medical Center combines consultations by rheumatologists and specialized rheumatology nurses (RNs). When indicated other specialists, such as gynecologists, will be involved.

In short, at each visit patients receive specialized care tailored to their reproductive goals from both a rheumatologist and a RN, addressing needs from preconception through six months post-delivery. The rationale behind our care pathway stems from our experience that in addition to medical consultations by rheumatologists, which mainly focus on disease and treatment strategies, complementary support is needed (6). The RNs play a pivotal role in addressing these holistic aspects of care, including emotional well-being, sexual health, work-life balance, and practical aspects of parenthood. Nursing consultations also include patient educational opportunities in relation to any concerns regarding pregnancy and rheumatic disease. The purpose of this coordinated approach between rheumatologists and RNs is to provide seamless support throughout every phase of the patient's reproductive journey, optimizing patient care outcomes.

Our primary objective was to evaluate patient satisfaction with our integrated care pathway. As secondary objectives we aimed to describe the problems patients experience during the care pathway process and to identify the unmet needs that were not covered by the care pathway. Ultimately, the results of this study can be used to determine the most effective timing and delivery of counseling information and to enhance the support provided to women with inflammatory arthritis throughout their journey into motherhood.

## Methods

### Patient population and data collection

We designed a customized questionnaire for women aged 18 years and older who had given birth between September 2018 and April 2021. These women, treated following the Reproductive Rheumatology care pathway at the Department of Rheumatology at Erasmus University Medical Center, were invited to fill in the questionnaire online. A reminder was sent to non-responders two months after the initial invitation.

### Questionnaire development

The customized questionnaire was structured into sections tailored to different phases around pregnancy: preconception, pregnancy, and postpartum. It covered topics such as family planning and family size, parenthood, social support received during these phases, and counseling provided by the care pathway team. Participants were also invited to provide suggestions and personal feedback through free-text options.

Additionally, the questionnaire included statements regarding women's perspectives on preconception, pregnancy, postpartum, parenthood, and support. Participants were asked to rate their agreement to these statements on a scale from “totally disagree” to “totally agree” (Likert scale). Lastly, participants rated their satisfaction with rheumatologists and RNs on a scale from 1 (lowest) to 10 (highest).

### Statistical analysis

Data analysis was performed using STATA version 17. Descriptive statistics were used to summarize demographic characteristics, satisfaction scores, and responses to questionnaire items. Categorical variables are presented as frequencies and percentages, while continuous variables are reported as means with standard deviations or as medians with interquartile ranges, depending on their distribution.

To assess differences in satisfaction scores between rheumatologists and specialized nurses, paired *t*-tests were conducted. Chi-square tests or Fisher's exact tests were used to evaluate associations between categorical variables, such as problems encountered during different pregnancy phases and their resolutions.

All statistical tests were two-sided, and *p*-values less than 0.05 were considered statistically significant.

### Ethics

This study was reviewed by the Erasmus University Medical Center ethics committee was deemed in compliance with the Helsinki declaration. All patients were 18 years or older, had a good understanding of the Dutch language and provided their informed consent by ticking a box on the front page of the online questionnaire.

## Results

Out of 181 women invited, 95 responded, resulting in a completion rate of 52.5%. The demographic characteristics of participants are presented in Table 1.

**Table 1:** Participants demographic and clinical characteristics at time of completing questionnaire.

	*N* = 95
Age, mean (range)	34.1 (26–46)
Diagnosis, *n* (%)	
Rheumatoid arthritis	39 (41)
Psoriatic arthritis	19 (20)
Spondyloarthritis	18 (19)
Juvenile idiopathic arthritis	16 (17)
Other immunologic disease	3 (3)
Disease duration, mean (SD)	
Rheumatoid arthritis	10.3 (6.5)
Psoriatic arthritis	6.6 (4.0)
Spondyloarthritis	6.9 (4.9)
Juvenile idiopathic arthritis	23.6 (5.3)
Other immunologic disease	7.0 (4.2)
Child(ren)	
Number, mean (range)	1.5 (1–3)
Age youngest child (months), mean (SD)	14.0 (7.6)
First child, *n* (%)	58 (61)
Housing situation, *n* (%)	
With partner and with children at home	86 (90.5)
Without partner and with children at home	1 (1)
Not specified	8 (8.5)
Education*	
Bachelor degree or higher, *n* (%)	58 (67)
Work*	
Paid work	
- *n* (%)	76 (87)
- Hours per week, mean (range)	27 (8–40)
Incapacitated for work (partially or complete), *n* (%)	6 (7)

* *n* = 87.

### Satisfaction with care

The results indicate high satisfaction among women with the care they received. They rated rheumatologists with an average score of 8.8/10 and specialized nurses with an average score of 9.2/10. Furthermore, 96% of participants (*n* = 80) reported consistent information across healthcare providers.

### Problems experienced during the care pathway

Seventy-five women (78.9%) did not experience any problems during the preconception, pregnancy, or postpartum phases. Among the problems reported by the remaining 18 women (19.4%), issues included managing disease flares (*n* = 13), difficulty conceiving (*n* = 4), lack of collaboration between specialists (*n* = 2), and other non-rheumatological issues (*n* = 1). Twelve women successfully resolved their problems with or without assistance from the care pathway team, while six women (6.4%) were unable to resolve their issues (pain/disease activity (*n* = 3), non-IA-related issues (*n* = 2) and concerns about safety of medication during pregnancy (*n* = 1).

Additionally, we examined whether being a first-time mother influenced the problems experienced. Of the six women who had unresolved issues, five were expecting their first child (data not shown).

### Information needs per phase (preconception, pregnancy and postpartum)

**Figure 1:**
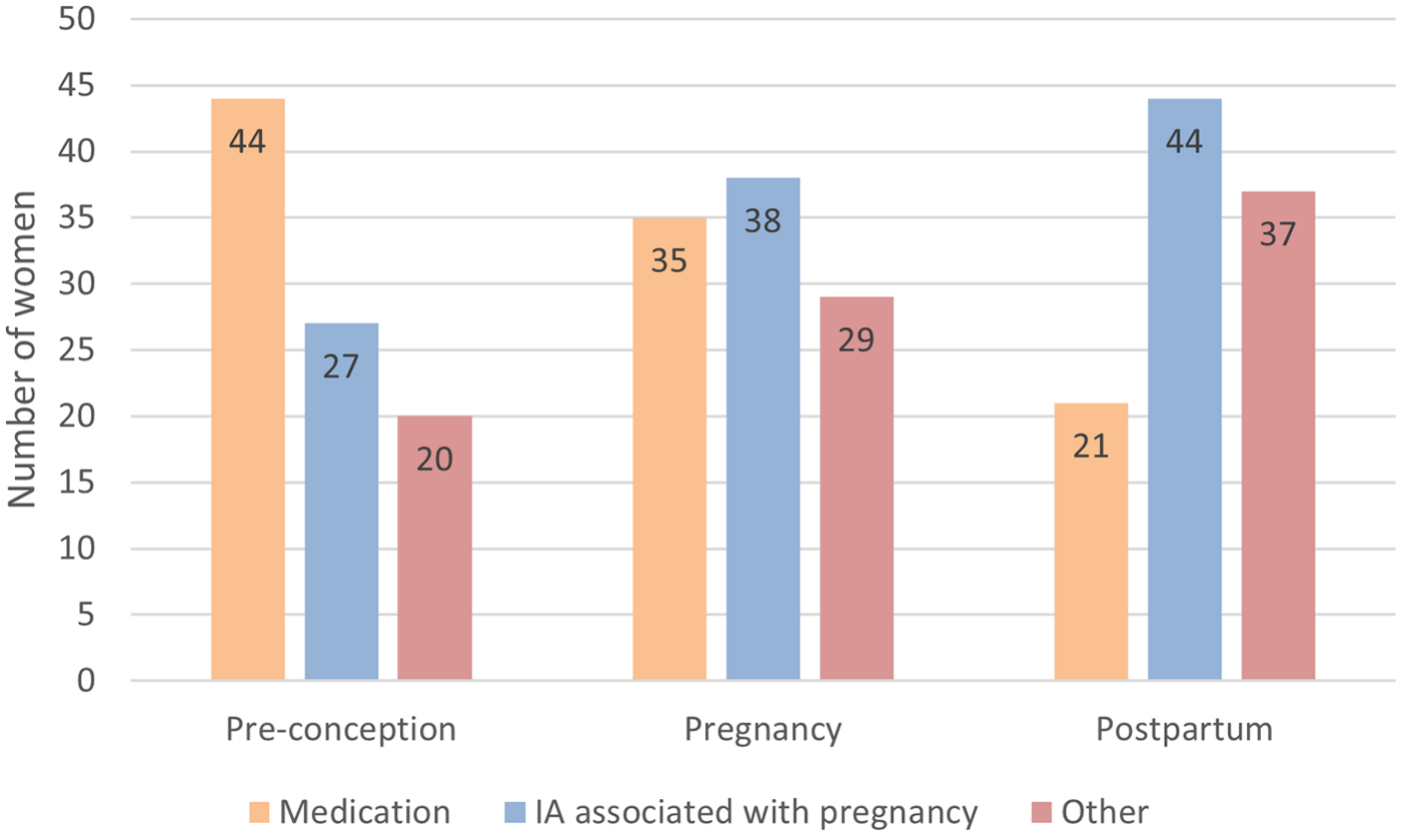
Topics women want to discuss in the different phases around pregnancy. *IA, inflammatory arthritis.

**Figure 2:**
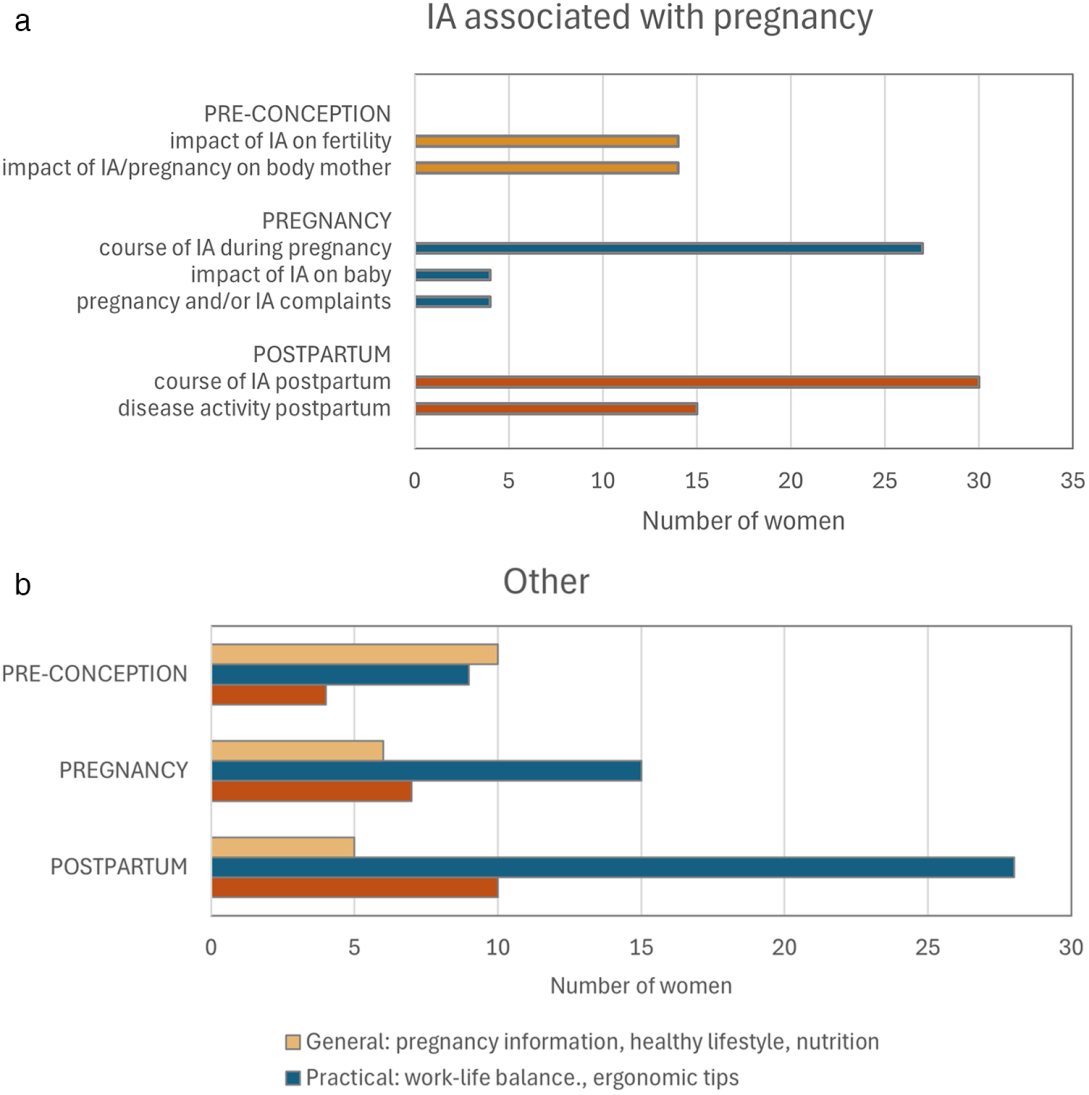
**(a)** Category “IA associated with pregnancy” specified. **(b)** Category “Other” specified.

During the preconception phase, participants primarily expressed a need for information about medication (Figure 1). They also needed details on how the disease and medication affect fertility and pregnancy, as well as general advice on lifestyle and nutrition. During pregnancy and after childbirth, the focus shifted to understanding the progression of the disease during these phases and practical advice on topics like breastfeeding, ergonomic tips and work-life balance. During the postpartum phase, more women expressed a desire for support from women in similar situations and other healthcare professionals (Figures 2a, b).

### Statements evaluation

Participants responded to statements regarding their perspectives on pregnancy, postpartum, parenthood, and support. Notably:

• “Through the information from the rheumatology care team, I gained more knowledge and understanding about, pregnancy, and the postpartum phase.” 88% of the women agreed or totally agreed with this statement.
• “I have difficulty accepting help.” Responses varied widely, with 50.6% indicating agreement or total agreement.
• “Through the rheumatology care team, I have become more confident about, pregnancy, and the postpartum phase.” Responses showed a positive trend, with 67.8% agreeing or totally agreeing.

## Discussion

Our study explored the experiences and expectations of women with IA accessing specialized care during pregnancy within the Reproductive Rheumatology care pathway. Overall, participants expressed high satisfaction with the integrated care provided by the care pathway, including rheumatologists and specialized nurses, highlighting the pathway's efficacy in meeting the complex needs of women with IA throughout their reproductive journey.

Challenges encountered, such as managing disease flares and difficulties with conception, were reported by a subset of participants. However, with the support of the care pathway team, most issues were effectively managed, highlighting the accessibility and effectiveness of healthcare interventions in this setting. The finding that a majority of women with unresolved problems were expecting their first child may indicate additional challenges for first-time mothers.

Information needs varied significantly across different pregnancy phases. During the preconception phase, participants sought guidance on medication safety, fertility, and hereditary considerations. In contrast, pregnancy and postpartum phases emphasized disease management, medication safety during breastfeeding, and overall well-being. Postpartum, there was also a strong desire for practical advice ergonomic tips, and shared experiences from other women.

Participants strongly endorsed the role of counseling provided by the care pathway team, reporting increased knowledge and confidence about, pregnancy, and postpartum care. However, challenges in accepting help and maintaining confidence were also noted, suggesting the importance of personalized support strategies tailored to individual needs.

Healthcare professionals should recognize that these factors can significantly influence a patient's overall well-being and should be integrated into clinical care. Additionally, it is important to encourage patients to feel comfortable seeking help, whether from healthcare providers, family, or their social circle. Creating a supportive atmosphere where patients feel empowered to ask for assistance is critical to improving both physical and emotional outcomes.

While our study benefited from a robust methodology, including a tailored questionnaire and high response rate, it is important to acknowledge limitations such as potential response bias, the retrospective nature of data collection and the use of a non-validated questionnaire. Future research could explore longitudinal outcomes and assess the sustained impact of interventions within similar specialized care pathways.

When comparing these findings to other studies on similar care pathways, our approach demonstrates the value of comprehensive integration between specialized nurses and rheumatologists, distinguishing it from traditional models that often lack coordinated care. For example, other studies have highlighted that fragmented care systems can leave patients feeling unsupported and overwhelmed (7, 8).

In contrast, our reproductive rheumatology model mitigates these issues by providing consistent, interdisciplinary, and holistic support throughout each phase of pregnancy. This includes flexible appointment scheduling, customized counseling, and a focus on psychological resilience, tailored to individual needs. These features not only improve patient confidence and acceptance of care but also contribute to enhanced outcomes and satisfaction. We believe these lessons could inform the development of more effective, patient-centered care pathways in the future.

In conclusion, our findings underscore the pivotal role of Reproductive Rheumatology care pathways in supporting women with IA through pregnancy. By addressing specific challenges and information needs, healthcare providers such as RNs can enhance the quality of care and outcomes for these women, ultimately improving their reproductive health and well-being.

## Data Availability

The raw data supporting the conclusions of this article will be made available by the authors, without undue reservation.

## References

[1] Smolen JS Aletaha D Barton A Burmester GR Emery P Firestein GS Rheumatoid arthritis. Nat Rev Dis Primers. (2018) 4:18001. 10.1038/nrdp.2018.1 29417936

[2] Smeele HTW Dolhain R. Current perspectives on fertility, pregnancy and childbirth in patients with rheumatoid arthritis. Semin Arthritis Rheum. (2019) 49(3S):S32–S5. 10.1016/j.semarthrit.2019.09.010 31779849

[3] Brouwer J Hazes JM Laven JS Dolhain RJ. Fertility in women with rheumatoid arthritis: influence of disease activity and medication. Ann Rheum Dis. (2015) 74(10):1836–41. 10.1136/annrheumdis-2014-205383 24833784

[4] Kemper E Ghalandari N Wintjes H Van Steensel-Boon A Kranenburg L Mulders A Active counselling and well-controlled disease result in a higher percentage of women with rheumatoid arthritis that breast feed: results from the PreCARA study. RMD Open. (2022) 8(2). 10.1136/rmdopen-2022-002194 35705306 PMC9204414

[5] Rotter T Kinsman L James E Machotta A Gothe H Willis J Clinical pathways: effects on professional practice, patient outcomes, length of stay and hospital costs. Cochrane Database Syst Rev. (2010) (3):CD006632. 20238347 10.1002/14651858.CD006632.pub2

[6] Primdahl J Sørensen J Horn HC Petersen R Hørslev-Petersen K. Shared care or nursing consultations as an alternative to rheumatologist follow-up for rheumatoid arthritis outpatients with low disease activity–patient outcomes from a 2-year, randomised controlled trial. Ann Rheum Dis. (2014) 73(2):357–64. 10.1136/annrheumdis-2012-202695 23385306

[7] Ingvarsson E Schildmeijer K Hagerman H Lindberg C. “Being the main character but not always involved in one’s own care transition” - a qualitative descriptive study of older adults’ experiences of being discharged from in-patient care to home. BMC Health Serv Res. (2024) 24(1):571. 10.1186/s12913-024-11039-3 38698451 PMC11067295

[8] Enthoven AC. Integrated delivery systems: the cure for fragmentation. Am J Manag Care. (2009) 15(10 Suppl):S284–90. 20088632

